# Cross-training between running and cycling: practitioner perspectives on transferability and exercise substitution

**DOI:** 10.3389/fspor.2026.1930728

**Published:** 2026-08-25

**Authors:** T. Menges, C. Dindorf, E. Bartaguiz, M. Fröhlich

**Affiliations:** 1 Endurance Coach GmbH, Berlin, Germany; 2 Department of Sports Science, RPTU University Kaiserslautern-Landau, Kaiserslautern, Germany

**Keywords:** cross-training, cycling, endurance training, exercise substitution, running, training frequency, training intensity, training load

## Abstract

**Background:**

Cross-training between running and cycling is widely used in endurance sports to maintain performance while managing training load and injury risk. However, experimental evidence regarding transfer effects between modalities remains inconsistent, and little is known about how athletes and coaches perceive and implement cross-training in practice.

**Objective:**

This study investigated how cross-training between running and cycling is perceived and applied by endurance athletes and coaches, with particular focus on direction-dependent transferability, training intensity, training frequency, and perceived equivalence durations.

**Methods:**

A cross-sectional online survey was conducted among 55 endurance athletes and coaches. Participants evaluated their perceptions of cross-training in both directions (running vs. cycling; cycling vs. running) using Likert-scale ratings and provided estimates of equivalent replacement durations between modalities. Differences between directions, intensities, and groups were analyzed using non-parametric procedures for ordinal single-item data and parametric analyses for reliable composite scores.

**Results:**

Participants perceived transferability between running and cycling to be direction-dependent, with a significant difference between substitution directions (*p* < 0.001). Participants estimated that replacing a running session with a cycling session would require substantially longer training durations (mean factor = 1.76; SD = 0.12), whereas shorter running sessions were considered sufficient to replace a cycling session (mean factor = 0.54; SD = 0.16). Participants estimates of equivalent replacement durations varied systematically across training durations and were not adequately represented by a constant conversion factor. Low-intensity training was perceived as significantly more transferable than threshold- or VO_2_max-oriented training (*p* < 0.001). Perceived training frequency adjustments also differed by direction, with cycling substitutions more often associated with maintained or increased training frequency. Overall, only minor differences between athletes, coaches, and individuals fulfilling both roles were observed.

**Conclusion:**

Cross-training between running and cycling was not perceived as a simple one-to-one replacement strategy, but rather as a direction-dependent and duration-dependent modification of training load. The findings suggest that perceived equivalence between modalities may not be adequately represented by fixed conversion ratios and that cross-training may be perceived as particularly suitable for low-intensity endurance training and high-volume load management.

## 1 Introduction

Cross-training refers to the systematic use of one exercise modality to maintain or enhance performance in another modality (1). It is widely applied in endurance sports to complement sport-specific training, reduce repetitive loading, and maintain physiological adaptations during periods of injury, recovery, or high training load. Among endurance disciplines, running and cycling represent one of the most common cross-training combinations because they share nearly similar aerobic demands while differing substantially in their mechanical loading characteristics (2).

Cross-training between running and cycling is widely implemented in endurance sports to maintain aerobic fitness while reducing sport-specific mechanical load. Running, for instance, is associated with high rates of overuse injuries due to repetitive impact forces, leading many athletes to incorporate lower-impact modalities such as cycling (3, 4). In addition, cross-training is commonly used to manage fatigue and reduce cumulative musculoskeletal stress during periods of high training load (5).

From a physiological perspective, cross-training assumes that central adaptations, such as improvements in VO_2_max, can transfer across exercise modalities involving large muscle groups (2). Despite a strong theoretical rationale, evidence for the transfer of training adaptations between running and cycling remains inconclusive. A recent meta-analysis reported no clear differences between modalities, with small effect sizes and substantial heterogeneity across studies (6). This variability limits firm conclusions and suggests that cross-training responses are influenced by multiple interacting factors that remain poorly understood. In particular, the potential role of transfer direction (running-to-cycling vs. cycling-to-running), as well as differences in training intensity, volume, and recovery demands, remains largely unexplored (7).

In addition, current research is predominantly based on controlled experimental settings and focuses on physiological outcomes (8–13), while largely neglecting how cross-training is applied in practice. In real-world training environments, athletes and coaches make practical decisions regarding the substitution of training modalities, considering not only performance but also fatigue, recovery, and training structure (14, 15). To our knowledge, no previous study has systematically investigated practitioners’ perceptions of cross-training between running and cycling, including perceived transferability, exercise substitution, and equivalent training durations. Addressing this gap may complement existing physiological research by providing insight into how cross-training is perceived and applied in practice.

Knowledge in sport science is derived not only from experimental research but also from practical experience accumulated through training and coaching practice (16). Whereas scientific evidence aims to establish generalizable principles under controlled conditions, practitioner knowledge reflects context-specific expertise developed through repeated application and evaluation in real-world environments (17). Integrating these practitioner perspectives may therefore provide valuable context for interpreting the current evidence base and informing future research.

Consequently, the present study integrates practitioner perspectives with the existing evidence base by examining how high-performance athletes and coaches perceive and apply cross-training between running and cycling. Using a structured questionnaire, the study addressed three research questions: i) Is the perceived transfer of training adaptations between running and cycling direction-dependent? (running vs. cycling or cycling vs. running) ii) How are training volume, frequency, and intensity adjusted when one modality is used to replace the other? and iii) Do these perceptions differ between athletes, coaches, and individuals fulfilling both roles?

By incorporating practitioner perspectives, this study contributes to a more nuanced understanding of cross-training between running and cycling and provides practical context for interpreting the current evidence base. The findings may help guide future research and support evidence-informed training prescription in endurance sports.

## 2 Methods

### 2.1 Study design and overview

This study used a cross-sectional online survey design to investigate how cross-training between running and cycling is perceived and implemented in endurance sports. Data were collected using a self-developed questionnaire (Table 1) administered via LimeSurvey (18) between December 10, 2025, and January 20, 2026. The survey was conducted as a one-time anonymous assessment without follow-up. Each participant was assigned a unique identifier to ensure data integrity while preserving confidentiality.

**Table 1:** Questionnaire items, response options and rationale for inclusion.

Item	Question	Why	Answer options
1	Scenario description	Introduces a standardized cross-training scenario to ensure all participants base their responses on the same assumptions.	–
2	How suitable is cycling training as a substitute for low-intensity running sessions?	Assesses perceived suitability of cycling as a replacement for low-intensity running.	Not suitable at all/Rather unsuitable/Partly suitable/Rather suitable/Very suitable
3	How should training duration be adjusted when a running session is replaced by a cycling session?	Assesses expert opinions regarding time-equivalent training load between modalities.	Much shorter/Somewhat shorter/Same duration/Somewhat longer/Much longer
4	To what extent should the number of weekly running sessions be transferred to cycling training?	Evaluates perceived equivalence of training frequency between running and cycling.	Significantly fewer sessions/Somewhat fewer sessions/Same number of sessions/Somewhat more sessions/Significantly more sessions
5a	To what extent can endurance-base running be replicated through cycling?	Assesses transferability of low-intensity aerobic adaptations.	Not suitable at all/Rather unsuitable/Partly suitable/Rather suitable/Very suitable
5b	To what extent can threshold running be replicated through cycling?	Assesses transferability of threshold-related adaptations.	Not suitable at all/Rather unsuitable/Partly suitable/Rather suitable/Very suitable
5c	To what extent can high-intensity (VO_2_max) running be replicated through cycling?	Assesses transferability of high-intensity aerobic adaptations.	Not suitable at all/Rather unsuitable/Partly suitable/Rather suitable/Very suitable
6	How effectively can physiological adaptations of running be achieved through cycling?	Evaluates the overall effectiveness of cycling for maintaining running-related adaptations.	Not effective at all/Rather ineffective/Partly effective/Rather effective/Very effective
7	Specify equivalent cycling durations for 30, 60, 120 and 180 min of low-intensity running.	Quantifies expert-derived time-conversion factors between running and cycling.	Open numerical response (minutes)
8	To what extent can running fitness be maintained by six weeks of cycling-only training?	Assesses expectations regarding long-term preservation of running performance through cycling.	Not at all/Rather not/Partly/Rather yes/Completely
9	Scenario description	Introduces the reverse transfer scenario (cycling replaced by running).	–
10	How suitable is running training as a substitute for low-intensity cycling sessions?	Assesses perceived suitability of running as a replacement for low-intensity cycling.	Not suitable at all/Rather unsuitable/Partly suitable/Rather suitable/Very suitable
11	How should training duration be adjusted when a cycling session is replaced by a running session?	Assesses perceived duration adjustments when transferring cycling load to running.	Much shorter/Somewhat shorter/Same duration/Somewhat longer/Much longer
12	To what extent should the number of weekly cycling sessions be transferred to running training?	Evaluates perceived equivalence of training frequency in the opposite transfer direction.	Significantly fewer sessions/Somewhat fewer sessions/Same number of sessions/Somewhat more sessions/Significantly more sessions
13a	To what extent can endurance-base cycling be replicated through running?	Assesses transferability of aerobic endurance adaptations from cycling to running.	Not suitable at all/Rather unsuitable/Partly suitable/Rather suitable/Very suitable
13b	To what extent can threshold cycling be replicated through running?	Assesses transferability of threshold adaptations from cycling to running.	Not suitable at all/Rather unsuitable/Partly suitable/Rather suitable/Very suitable
13c	To what extent can high-intensity (VO_2_max) cycling be replicated through running?	Assesses transferability of high-intensity adaptations from cycling to running.	Not suitable at all/Rather unsuitable/Partly suitable/Rather suitable/Very suitable
14	How effectively can physiological adaptations of cycling be achieved through running?	Evaluates the overall effectiveness of running for maintaining cycling-related adaptations.	Not effective at all/Rather ineffective/Partly effective/Rather effective/Very effective
15	Specify equivalent running durations for 45, 60, 120 and 180 min of low-intensity cycling.	Quantifies expert-derived conversion factors from cycling to running.	Open numerical response (minutes)
16	To what extent can cycling fitness be maintained by six weeks of running-only training?	Assesses expectations regarding long-term preservation of cycling performance through running.	Not at all/Rather not/Partly/Rather yes/Completely
17	Introduction to graphical conversion models.	Ensures participants understand the presented conversion-factor concepts before evaluation.	–
18	How plausible is an increasing conversion factor from cycling to running with longer low-intensity training duration?	Assesses agreement with a duration-dependent transfer model from cycling to running.	Strongly disagree/Disagree/Neutral/Agree/Strongly agree
19	If applicable, suggest modifications to the presented conversion factor.	Collects qualitative expert feedback to refine the proposed model.	Open response
20	How plausible is a decreasing conversion factor from running to cycling with longer low-intensity training duration?	Assesses agreement with a duration-dependent transfer model from running to cycling.	Strongly disagree/Disagree/Neutral/Agree/Strongly agree
21	If applicable, suggest modifications to the presented conversion factor.	Collects qualitative expert feedback to refine the proposed model.	Open response
22	Cycling intensity should be prescribed using cycling-specific heart-rate zones rather than running zones.	Assesses consensus regarding modality-specific intensity prescription and acknowledges known physiological differences in heart-rate responses between sports.	Strongly disagree/Disagree/Neutral/Agree/Strongly agree
23	Cyclists generally perform fewer but longer sessions than runners.	Assesses expert agreement regarding discipline-specific training structure and distribution of training volume.	Strongly disagree/Disagree/Neutral/Agree/Strongly agree
24	From five weekly sessions onwards, an additional endurance sport should be incorporated.	Assesses perceptions regarding the value of complementary endurance training and cross-training integration.	Strongly disagree/Disagree/Neutral/Agree/Strongly agree
25	If applicable, what should the ratio between primary and secondary sport be?	Identifies expert recommendations regarding the optimal distribution between sport-specific and cross-training volume.	50:50/60:40/70:30/80:20/90:10
26	Are you an athlete, a coach, or both?	Characterizes respondent role and source of expertise.	Athlete/Coach/Athlete & Coach
27	In which sport are you active as an athlete or coach?	Identifies the respondent's sport-specific background and perspective.	Cycling/Running/Cycling & Running/Duathlon/Triathlon
28	How many years have you been an athlete and/or coach?	Quantifies practical experience relevant to the topic.	Open numerical response (years)
29	Do you hold a coaching certification?	Assesses formal coaching qualifications.	Yes/No
30	If yes, which coaching certification do you hold?	Provides additional information regarding professional coaching expertise.	Open response
31	In which age category do you or your athletes compete?	Characterizes the athlete population represented by the respondent.	Youth & Junior/U23/23–29 years/30–40 years/>40 years
32	Are you involved in high-performance sport?	Differentiates respondents according to performance level and training environment.	Yes/No
33	At which competition level do you or your athletes primarily compete?	Characterizes the competitive context and expertise level of the respondent.	Amateur/Regional/State/National/International

The questionnaire was specifically developed based on the findings of the preceding meta-analysis (6) and aimed to capture applied perspectives on cross-training between running and cycling. All participants completed the same questionnaire under standardized conditions to ensure comparability across groups.

### 2.2 Participants and recruitment

Participants were recruited from performance-oriented endurance sports, including triathlon, cycling, and running, ranging from youth to adult competitive levels. Recruitment was conducted through personal and professional networks, regional and national sport federations in Germany, Austria, and Switzerland, and high-performance training centers, including the Olympic Training Center Rhineland-Palatinate and Saarland. National training centers were contacted by e-mail and asked to distribute the survey among their coaches and competitive athletes. This recruitment procedure resulted in a snowball sampling approach, as coaches further distributed the survey to athletes within their professional networks.

Eligible participants were athletes, coaches, or individuals fulfilling both roles, provided they self-identified as being involved in high-performance sport. Participants were additionally required to report the highest level at which they competed or coached (national or international). Coaches were additionally required to hold a valid coaching license (Level A, B, or C, or Diploma Coach). Participants were excluded if they reported only recreational sport involvement or if the questionnaire was incomplete. Of the 97 individuals who initiated the survey, 55 provided complete responses and were included in the final analysis, whereas 42 responses were excluded due to incomplete data. An *a priori* sample size calculation was conducted using G*Power 3.1 for a paired comparison of two dependent ratings, reflecting the primary within-subject comparison between running-to-cycling and cycling-to-running substitution. As no prior data were available for perceived cross-training transferability between running and cycling, the expected effect size was approximated from previously reported coach–athlete differences in perceived low-intensity training load. Inoue et al. (2022) (15) reported small-to-moderate differences for easy-session RPE and sRPE (SMD = 0.44–0.54), while no consistent differences were observed for moderate or hard sessions. Therefore, a medium effect size of *d* = 0.45 was assumed. With *α* = 0.05 and power = 0.90, the required sample size was 54 participants. The final sample of 55 complete responses met this requirement.

Participants represented performance-oriented endurance sports disciplines, primarily triathlon (*n* = 42), with additional representation from running (*n* = 7) and cycling (*n* = 6). The sample was not intended to proportionally represent different endurance disciplines. Instead, recruitment deliberately focused on triathletes, as they frequently replace running sessions with cycling, or vice versa, for example as part of training planning, injury management, or load regulation. Therefore, they were considered particularly well suited to evaluate perceived cross-training transferability and exercise substitution between the two modalities. The final sample consisted of 24 athletes, 21 coaches, and 10 participants who identified as both athlete and coach. Participants ranged from youth to adult performance levels and were involved in various competition formats within endurance sports. The average experience was 11 years for athletes, 12.3 years for coaches, and 21 years for participants with dual roles. Among the coaches, nine held a Level A license, five a Level B license, three a Level C license, and four were Diploma Coaches. Among participants fulfilling both roles, two held a Level A license, two a Level B license, and six a Level C license.

### 2.3 Questionnaire

The primary variables of interest were perceived transferability and practical implementation of cross-training between running and cycling. These variables were assessed separately for both substitution directions (running-to-cycling and cycling-to-running). Participants evaluated multiple training-related dimensions, including perceived suitability of cross-training, adjustments in training duration and session frequency, transferability of different intensity domains (low-intensity, threshold, and VO_2_max oriented training), and the effectiveness of cross-training for maintaining sport-specific physiological adaptations. In addition, participants estimated equivalent training durations for regenerative sessions in the alternate modality.

As no existing questionnaire addressing perceived cross-training transferability between running and cycling was available, the questionnaire was specifically developed for the present study based on the available literature and exploratory interviews with national triathlon coaches. These interviews were conducted to explore how coaches substitute running and cycling sessions in practice and to identify the variables they considered most relevant for evaluating cross-training transferability and exercise substitution between the two modalities. The preliminary questionnaire was subsequently reviewed by researchers (CD & MF) from the Department of Sport Science at RPTU University Kaiserslautern-Landau, Kaiserslautern, Germany to evaluate the relevance, clarity, and comprehensiveness of the items and to establish content validity. Finally, the questionnaire was pilot tested with five competitive endurance athletes to assess the clarity and comprehensibility of the questionnaire. Based on their feedback, minor wording revisions were made before the final version was administered.

Responses were primarily collected using Likert-type scales (19) ranging from 1 to 5. Depending on the specific item, verbal anchors reflected different ordered response categories, such as “not effective” to “very effective,” “much shorter” to “much longer,” or “strongly disagree” to “strongly agree”. In addition to ordinal response formats, some variables were recorded as continuous free-text numerical estimates, particularly for equivalent training durations expressed in minutes. Participant characteristics, including role (athlete, coach, or both), sport, experience, coaching qualification, years of expertise, performance level, and competition level, were collected using categorical and open-ended questions.

To assess perceived substitution between running and cycling, two conceptually related item sets were defined *a priori*. The running-to-cycling substitution construct comprised Items 2, 5a, 6, and 8, addressing perceived suitability for regenerative running replacement, transferability of low-intensity endurance adaptations, maintenance of physiological adaptations, and longer-term substitution of running through cycling. The cycling-to-running substitution construct comprised the corresponding Items 10, 13a, 14, and 16.

Internal consistency was evaluated using Cronbach's alpha. The running-to-cycling item set demonstrated acceptable reliability (*α* = 0.75), and a composite score was therefore calculated by averaging the four items. In contrast, the cycling-to-running item set showed limited internal consistency (*α* = 0.55). Additional reliability analyses indicated weak-to-moderate inter-item correlations (r = 0.09–0.38; mean r = 0.25), corrected item-total correlations ranging from r = 0.23 to r = 0.47, and no meaningful improvement in reliability when individual items were removed. Consequently, no composite score was calculated for the cycling-to-running construct, and all items were analyzed separately.

### 2.4 Statistical analysis

All statistical analyses were conducted using R (RStudio) (20). Descriptive statistics were calculated for all variables.

Single Likert-type items were treated as ordinal variables and analyzed using non-parametric statistical procedures. Differences between paired conditions, such as running-to-cycling and cycling-to-running transfer ratings, were examined using Wilcoxon signed-rank tests. Group differences between athletes, coaches, and athlete–coach individuals were assessed using Kruskal–Wallis tests. Where significant effects were detected, *post-hoc* pairwise comparisons were performed using Mann–Whitney *U* tests with Holm correction for multiple testing.

To investigate repeated-measures differences across multiple intensity domains or training durations, Friedman tests were conducted. Significant Friedman tests were followed by paired Wilcoxon signed-rank *post-hoc* tests with Holm correction.

Difference scores were additionally calculated to quantify directional preferences between transfer directions. Continuous variables, such as estimated equivalent training durations, were analyzed descriptively. Only complete questionnaires were included in the analyses. Participants who did not complete the questionnaire were excluded prior to statistical analysis. Consequently, no missing item-level data were present in the final dataset. Statistical significance was set at *p* < 0.05.

#### 2.4.1 Perceived substitution (item set for each direction)

For the running-to-cycling substitution construct a composite score was calculated by averaging the four items because the item set demonstrated acceptable internal consistency (Cronbach's *α* = 0.75). Group differences in the composite score were examined using one-way analysis of variance (ANOVA). Normality was assessed using Shapiro–Wilk tests and homogeneity of variance using Levene's test. Although deviations from normality were observed in two groups, variance homogeneity was satisfied [Levene's test: F(2,52) = 2.01, *p* = 0.145]. Given the acceptable reliability of the composite score and the robustness of ANOVA to moderate violations of normality, parametric procedures were considered appropriate.

For the cycling-to-running substitution construct, no composite score was calculated because the item set demonstrated insufficient internal consistency (Cronbach's *α* = 0.55), which was below the predefined threshold for scale construction. Consequently, individual Likert-scale items were analyzed separately using non-parametric procedures.

## 3 Results

### 3.1 Outcome data

#### 3.1.1 Perceived substitution of running by cycling

**Figure 1:**
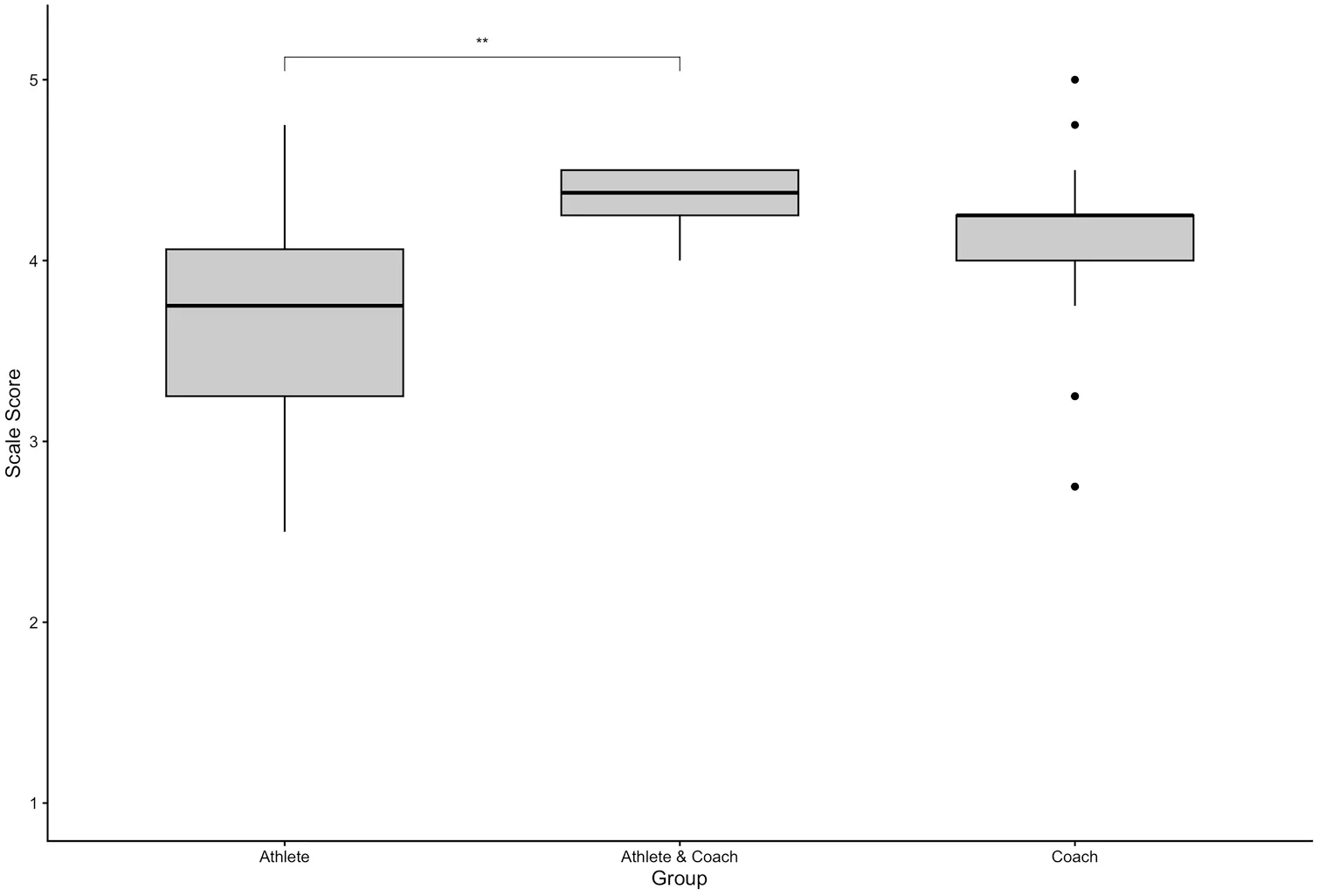
Substitution of running by cycling. **Means significant <0.05.

ANOVA results show a significant effect of group membership on substitution ratings [F (2,52) = 5.48, *p* = 0.007, *η^2^* = 0.17]. *Post-hoc* Tukey analyses revealed a significant difference between athletes and athlete–coach individuals [d = 1.18, 95% CI (0.39, 1.97), *p* = 0.008], whereas no significant differences were observed between athletes and coaches [d = 0.64, 95% CI (0.03, 1.26), *p* = 0.089] or between coaches and athlete–coach individuals [d = 0.54, 95% CI (0.24, 1.31), *p* = 0.35] (Figure 1).

#### 3.1.2 Perceived substitution of cycling by running

**Figure 2:**
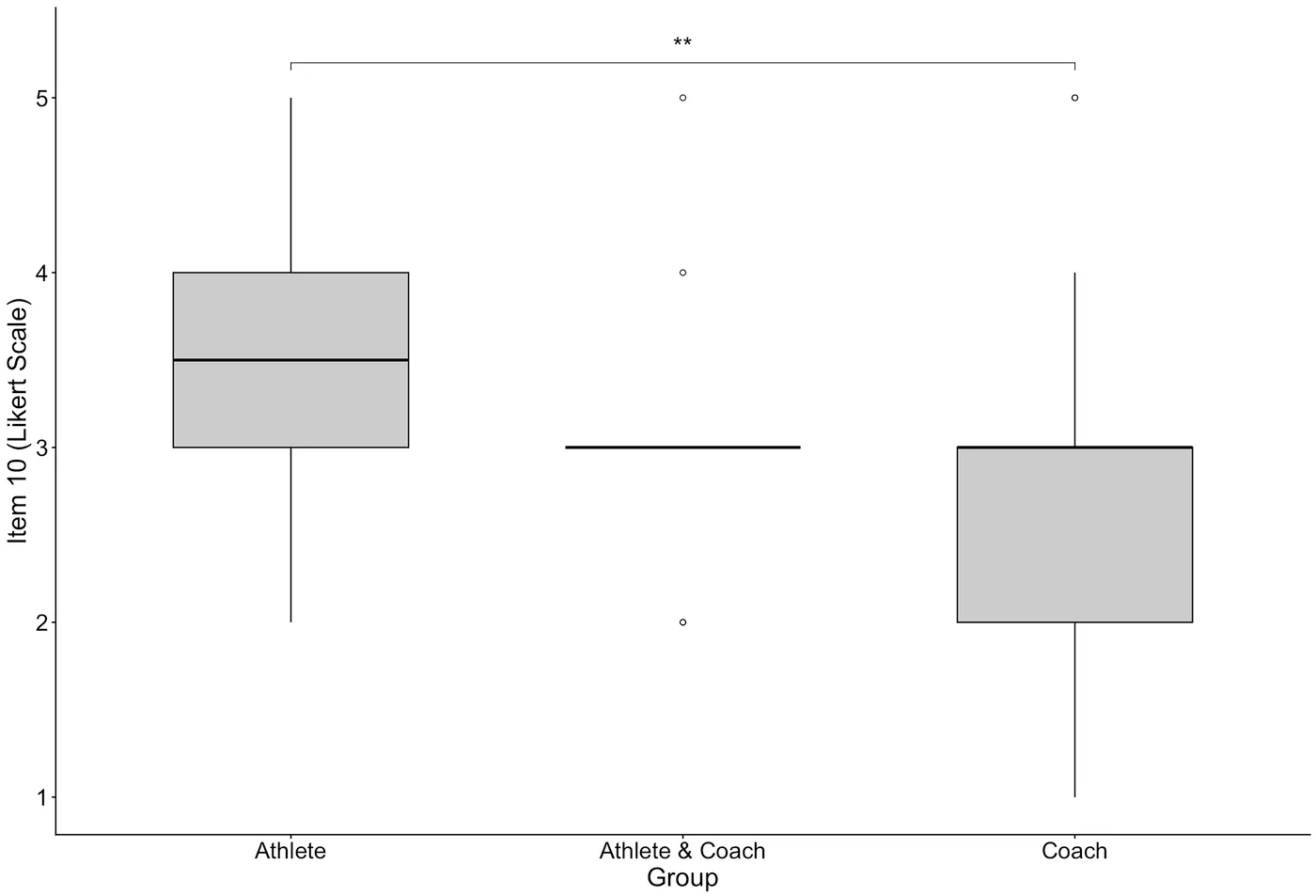
Substitution of cycling by running. Differences between groups. **Means significant <0.05.

A significant group effect was observed only for Item 10 [*χ*^2^ (2) = 9.36, *p* = 0.009, *η^2^* = 0.14]. *Post-hoc* analyses revealed a significant difference between athletes and coaches (*p* = 0.012, *r* = 0.43), while the remaining group comparisons were not significant (Figure 2). Descriptive boxplot analyses indicated that coaches generally rated running as a more suitable substitute for cycling compared to athletes, while the athlete–coach group showed intermediate values.

No significant group differences were observed for Item 13 [*χ*^2^ (2) = 0.79, *p* = 0.67], Item 14 [*χ*^2^ (2) = 1.24, *p* = 0.53], and Item 16 [*χ*^2^ (2) = 3.48, *p* = 0.17].

Overall, the results indicate that perceptions differed between groups only for the regenerative substitution aspect captured in Item 10, whereas evaluations of the remaining cycling-to-running transfer dimensions were largely comparable across groups.

#### 3.1.3 Direct comparison of training directions

A paired Wilcoxon signed-rank test comparing Item 2 (“How suitable is cycling training as a substitute for running training?”) and Item 10 (“How suitable is running training as a substitute for cycling training?”), which explicitly assessed the two transfer directions, showed a significant difference between running-to-cycling and cycling-to-running ratings (V = 934, *p* < 0.001, *r* = 0.71), indicating systematic differences in perceived transfer between both directions.

Running-to-cycling substitution received a median rating of 5, whereas cycling-to-running substitution received a median rating of 3. The estimated location shift was 1.50, 95% CI [1.00, 2.00].

**Figure 3:**
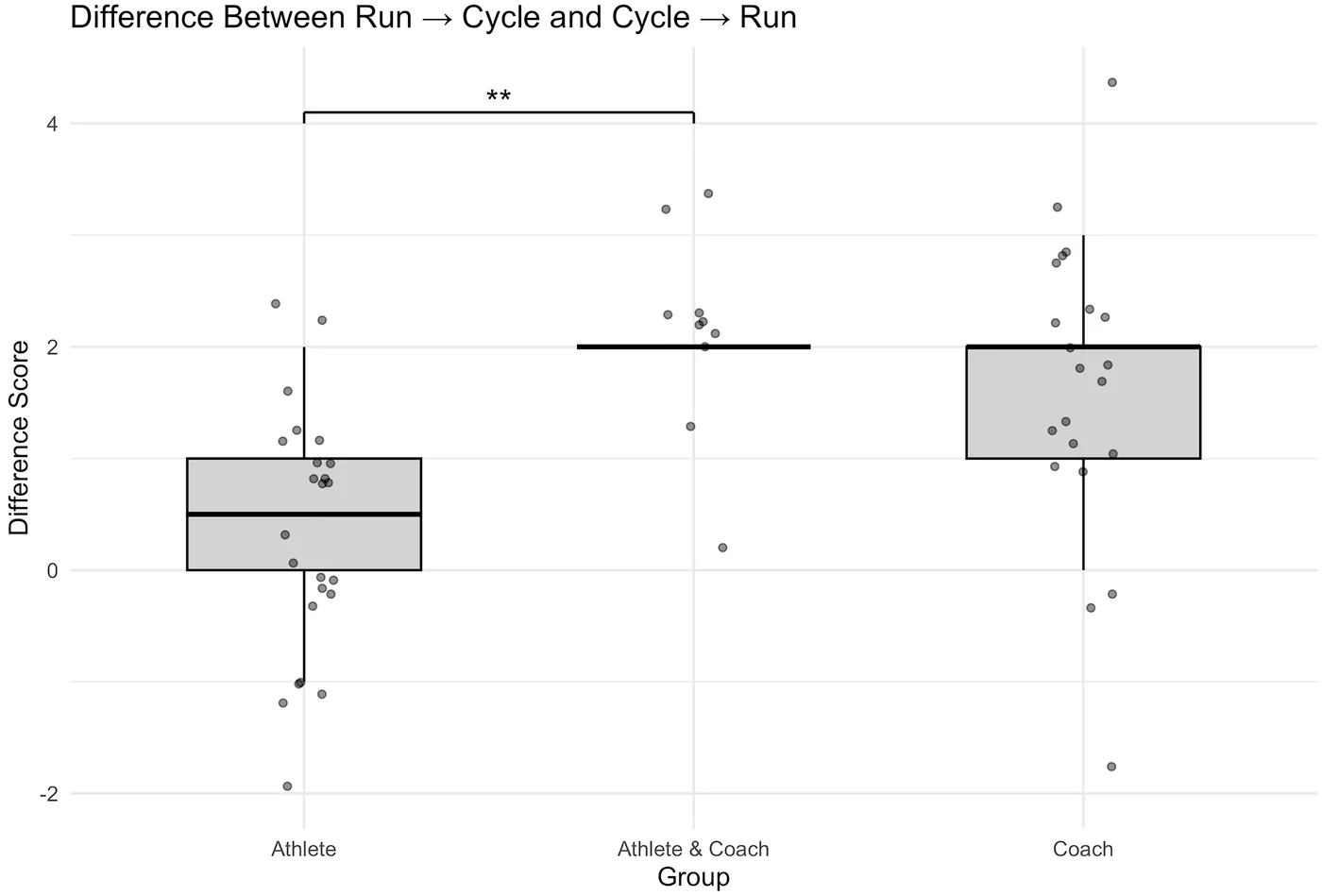
Differences between transfer directions. **Means significant <0.05.

Further analysis of difference scores revealed significant group differences [Kruskal–Wallis *χ*^2^ (2) = 16.62, *p* < 0.001, *ε^2^* = 0.28]. *Post-hoc* comparisons indicated that athletes differed significantly from both coaches (*p* = 0.002, *r* = 0.49) and athlete–coaches (*p* = 0.002, *r* = 0.58), while no significant difference was observed between coaches and athlete–coach individuals (*p* = 0.549, *r* = 0.11) (Figure 3). Boxplot visualization indicated group-specific differences in difference scores.

#### 3.1.4 Perceptions of cross-training implementation

**Figure 4:**
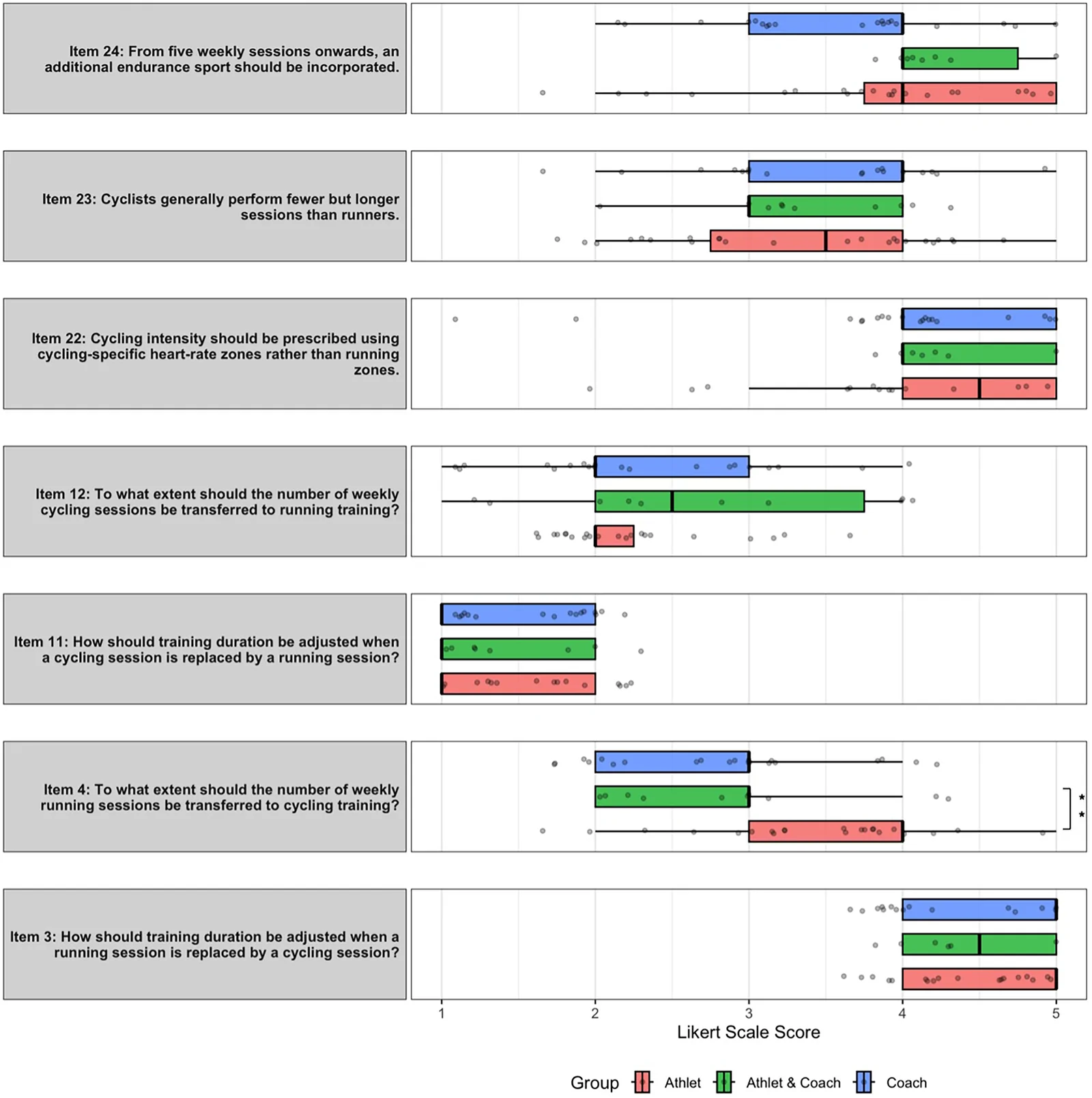
Items focusing on adjustments in training duration, session frequency, intensity regulation, and the general integration of cross-training. **Means significant <0.05.

Kruskal–Wallis tests revealed a significant group difference only for Item 4 [*χ*^2^ (2) = 9.46, *p* = 0.009, *ε^2^* = 0.14]. All other items showed no statistically significant group differences (all *p* > 0.05). Descriptive analyses and boxplot visualizations nevertheless revealed distinct response patterns across items (Figure 4).

Across all items, descriptive patterns indicated consistently higher agreement and lower variability for items related to intensity regulation (Item 22) and the integration of cross-training at higher training frequencies (Item 24). In contrast, greater variability was observed among athletes compared to coaches, while responses within the coach group were generally more homogeneous. Additionally, systematic differences between substitution directions were evident, particularly for training duration, with participants recommending longer durations when substituting running with cycling (Item 3) and shorter durations when substituting cycling with running (Item 11).

#### 3.1.5 Equivalent training duration

To investigate perceived equivalence between regenerative running and cycling sessions, participants estimated how many minutes of one modality would be required to replace predefined durations of the other modality while maintaining a comparable regenerative training effect. Four replacement durations were assessed in each direction using free-text responses (running vs. cycling and cycling vs. running).

The analyses addressed four primary research topics:

##### 3.1.5.1 Differences between replacement directions

To examine whether participants perceived the replacement of running by cycling differently from the replacement of cycling by running, equivalence factors were first calculated for each substitution scenario as ratios between the estimated replacement duration and the original training duration:

- Running vs. cycling:
  Factor = cycling duration/running duration
- Cycling vs. running:
  Factor = running duration/cycling duration

Subsequently, participant-specific mean equivalence factors were calculated separately for both directions. These aggregated direction-specific estimates were then compared using a paired Wilcoxon signed-rank test. A paired *t*-test was not applied because the Shapiro–Wilk test indicated a violation of the normality assumption for the paired difference scores (*p* < 0.05).

The Wilcoxon signed-rank test revealed a highly significant difference between substitution directions (V = 1540, *p* < 0.001, *r* = 0.87). The Hodges–Lehmann estimate indicated a median paired difference of 1.21 [95% CI (1.09, 1.34)], confirming that participants consistently perceived the replacement of running by cycling differently from the replacement of cycling by running. From a practical perspective, this indicates that practitioners perceive different duration adjustments depending on the direction of substitution.

The calculated equivalence factors differed substantially between directions. For cycling vs. running, the mean factor was 0.55 whereas for running vs. cycling, the mean factor was 1.76 (Table 2).

**Table 2:** Equivalence factors.

Direction	*n*	Mean factor	SD	Median factor	IQR
Cycling vs. Running	55	0.54	0.16	0.51	0**.**11
Running vs. Cycling	55	1.76	0.36	1.82	0**.**47

Across all durations, the running vs. cycling factors were consistently greater than 1 (e.g., mean = 2.01 for 30 min running), whereas the cycling vs. running factors remained consistently below 1 (mean = 0.48–0.57 depending on duration) (Supplementary Table 5).

##### 3.1.5.2 Do the groups differ in their estimations?

To investigate whether athletes, coaches, and participants identifying as both athlete and coach differed in their replacement estimations, separate Kruskal–Wallis tests were conducted for each replacement duration. Shapiro–Wilk tests revealed significant violations of normality assumptions for the free-text estimation variables (all *p* < 0.001). Therefore, non-parametric procedures were applied. Significant effects were followed by pairwise Wilcoxon rank-sum tests with Holm correction for multiple comparisons.

Group differences were limited and only observed for short-duration substitutions in the running-to-cycling direction:

- 30 min running vs. cycling: *χ*^2^ (2) = 7.44, *p* = 0.024, *ε^2^* = 0.10
- 60 min running vs. cycling: *χ*^2^ (2) = 7.24, *p* = 0.027, *ε^2^* = 0.10

*Post-hoc* analyses revealed significant differences between athletes and coaches for both durations (30 min: *p* = 0.023, *r* = 0.40; 60 min: *p* = 0.037, *r* = 0.37), with athletes reporting higher equivalent cycling durations than coaches.

No significant group differences were observed:

- for longer durations (≥120 min),
- for any cycling vs. running substitutions,
- or for comparisons involving the athlete–coach group.

In addition to group comparisons, descriptive measures of dispersion (standard deviation, interquartile range), and range (Supplementary Table 6) were examined to assess the consistency of estimations within groups. Responses showed greater variability for short and medium running-to-cycling substitutions, particularly among athletes, whereas coaches generally provided more homogeneous estimations. Overall, group differences were limited despite some variation in within-group agreement across replacement durations and substitution directions.

##### 3.1.5.3 What equivalence durations were assumed?

To derive practically applicable equivalence values between regenerative running and cycling sessions, descriptive statistics (mean, median, standard deviation, interquartile range, minimum, and maximum) were calculated for each replacement scenario (Supplementary Table 7). Because several variables showed skewed distributions and occasional extreme responses, median values were interpreted as the most representative estimates of perceived equivalence.

Running vs. Cycling

Participants estimated the following equivalent cycling durations for replacing regenerative running sessions (Table 3).

**Table 3:** Equivalent cycling duration.

Running duration	Mean cycling duration	SD	Median cycling duration	IQR
30 min running	60.2 min	13.8	60 min	40
60 min running	108 min	20.8	120 min	70
120 min running	201 min	62.7	210 min	0
180 min running	282 min	99.7	300 min	0

Cycling vs. Running

Participants estimated the following equivalent running durations for replacing regenerative cycling sessions (Table 4).

**Table 4:** Equivalent running duration.

Cycling duration	Mean running duration	SD	Median running duration	IQR
45 min cycling	25.6 min	8.94	25 min	15
60 min cycling	39.5 min	29.7	30 min	20
120 min cycling	58.7 min	25.0	60 min	0
180 min cycling	86.5 min	26.8	90 min	0

**Figure 5:**
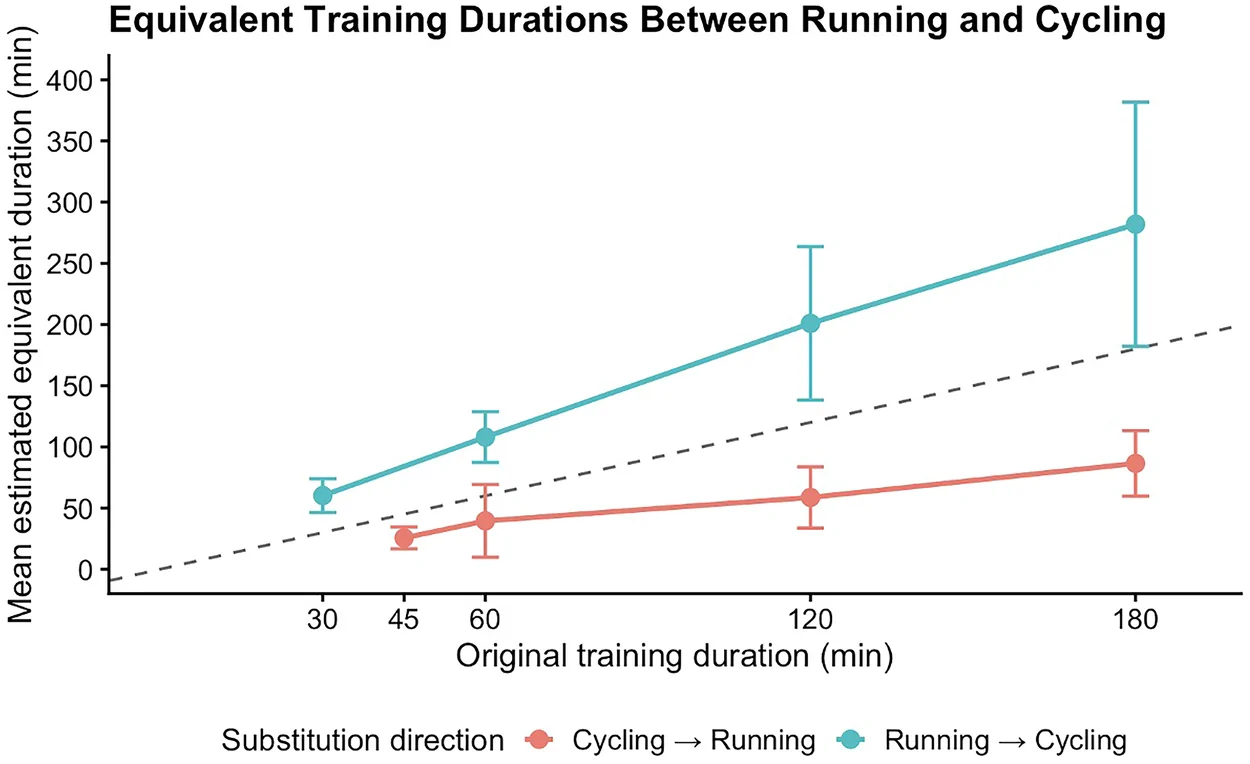
Mean estimated equivalent training durations for running-to-cycling and cycling-to-running substitutions. Error bars represent standard deviations (SD). The dashed line indicates a 1:1 substitution.

Overall, the descriptive results indicate that participants perceived substantially longer cycling durations to be necessary to replace regenerative running sessions, whereas shorter running durations were generally considered sufficient to replace cycling sessions (Figure 5).

##### 3.1.5.4 Are regenerative running and cycling linearly interchangeable?

To examine whether the equivalence durations between regenerative running and cycling changed across different training durations, the calculated equivalence factors were compared between durations using Friedman tests for repeated measures.

For the running-to-cycling direction, the Friedman test revealed significant differences in the calculated equivalence factors across durations [*χ*^2^ (3) = 46.90, *p* < 0.001, Kendall's *W* = 0.28]. Pairwise Wilcoxon signed-rank tests with Holm correction indicated significant differences between all duration pairs (all *p* ≤ 0.034), suggesting that the equivalence factor decreased systematically as the original running duration increased. Effect sizes ranged from moderate to large (*r* = 0.44–0.85; Supplementary Tables 8, 9).

Similarly, for the cycling-to-running direction, the Friedman test also revealed significant differences across durations [*χ*^2^ (3) = 49.19, *p* < 0.001, Kendall's *W* = 0.30]. *Post-hoc* analyses showed significant differences between all duration pairs except the comparison between 120 and 180 min (*p* = 0.363), indicating that the equivalence factor stabilized at longer exercise durations. Effect sizes ranged from small to large (*r* = 0.17–0.88; Supplementary Tables 8, 9).

Overall, participants did not perceive regenerative running and cycling as linearly interchangeable.

To further describe the relationship between duration and equivalence, logarithmic functions were fitted:

- Running vs. cycling: f(x) = −0.23885 · ln(x) + 2.81102
- Cycling vs. running: f(x) = −0.0959 · ln(x) + 0.9129

From a practical perspective, the logarithmic equations allow the calculation of duration-specific adjustment factors for exercise substitution, indicating that the required increase or decrease in training duration depends on the duration of the original training session rather than being represented by a single fixed factor.

#### 3.1.6 Group × direction × intensity analysis

**Figure 6:**
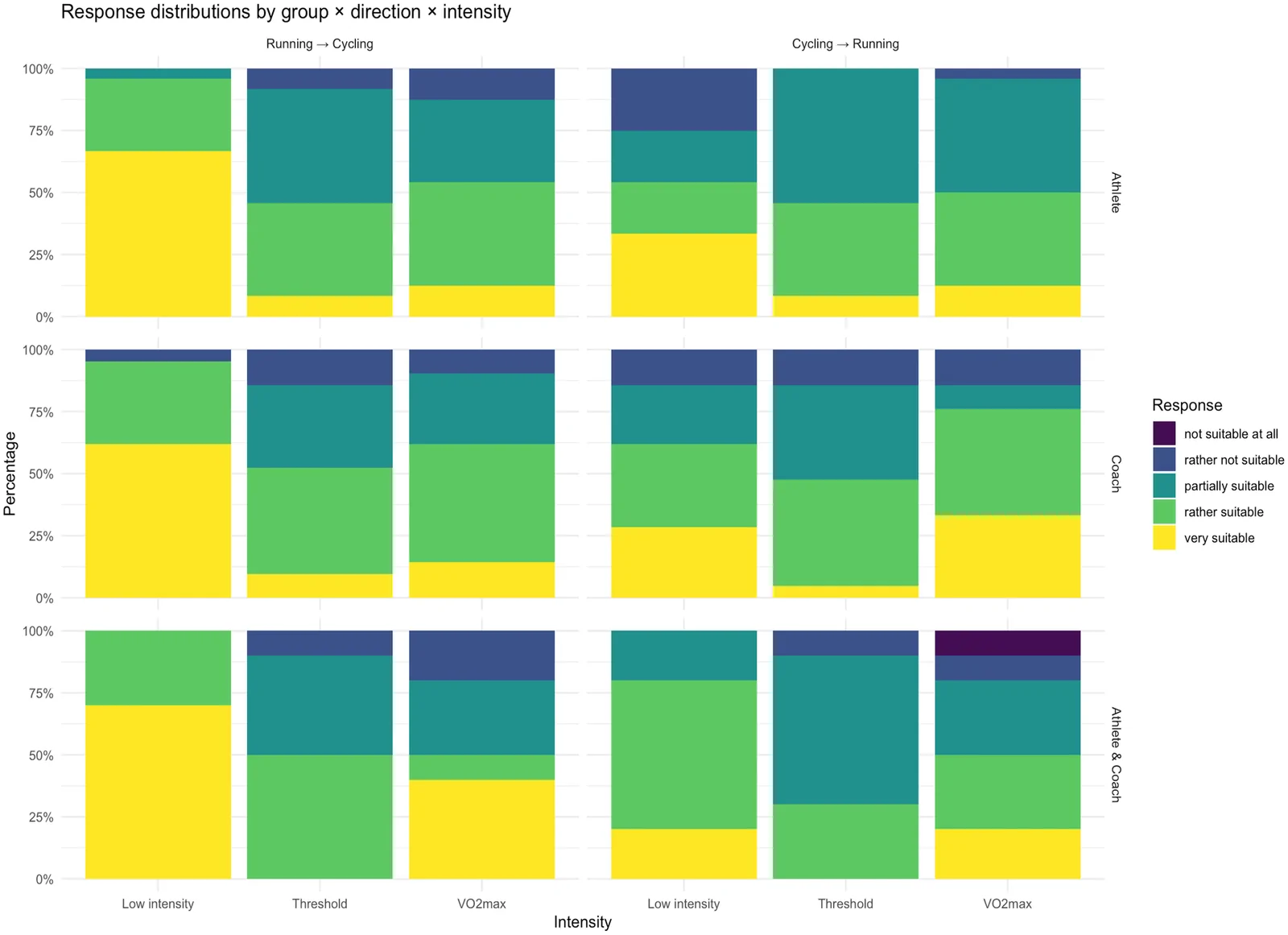
Percentage distribution of suitability ratings across participant groups, substitution directions, and training intensities. Colors indicate the proportion of responses in each of the five response categories, ranging from “not suitable at all” to “very suitable”. Each stacked bar represents the percentage distribution of responses for one intensity level within a specific group and substitution direction.

To investigate whether perceived transferability between running and cycling differed depending on training intensity, transfer direction, and participant group, non-parametric analyses were conducted separately for each factor combination (Figure 6). The analyses included three participant groups (athletes, coaches, athlete–coaches), two transfer directions (running vs. cycling; cycling vs. running), and three intensity domains (low-intensity, threshold, VO_2_max). Since the data were based on ordinal Likert-scale ratings, non-parametric procedures were applied throughout.

##### 3.1.6.1 Group differences

Potential group differences were examined using separate Kruskal–Wallis tests for each combination of transfer direction and training intensity.

No significant group differences were observed for any combination of direction and intensity:

- Running vs. Cycling (Low-intensity): *χ*^2^ (2) = 0.28, *p* = 0.86
- Running vs. Cycling (Threshold): *χ*^2^ (2) = 0.06, *p* = 0.97
- Running vs. Cycling (VO_2_max): *χ*^2^ (2) = 0.30, *p* = 0.86
- Cycling vs. Running (Low-intensity): *χ*^2^ (2) = 0.54, *p* = 0.76
- Cycling vs. Running (Threshold): *χ*^2^ (2) = 1.35, *p* = 0.50
- Cycling vs. Running (VO_2_max): *χ*^2^ (2) = 3.09, *p* = 0.21

After Holm correction, all adjusted *p*-values remained non-significant.

##### 3.1.6.2 Differences between transfer directions

To compare the two transfer directions, participant ratings were aggregated across intensities using median values and analyzed using a paired Wilcoxon signed-rank test. The Wilcoxon signed-rank test revealed a significant difference between transfer directions (V = 216, *p* = 0.045, *r* = 0.41).

##### 3.1.6.3 Differences between intensities

To examine whether perceived transferability differed across intensity domains (Low-intensity, threshold, VO_2_max), separate Friedman tests for repeated measures were conducted for each transfer direction. Significant Friedman tests were followed by paired Wilcoxon signed-rank *post-hoc* tests with Holm correction.

Running vs. Cycling

For the running vs. cycling direction, the Friedman test revealed significant differences between intensity domains [*χ*^2^ (2) = 57.31, *p* < 0.001, Kendall's *W* = 0.52].

*Post-hoc* analyses showed:

- Low-intensity vs. Threshold: *p* < 0.001, *r* = 0.84
- Low-intensity vs. VO_2_max: *p* < 0.001, *r* = 0.76
- Threshold vs. VO_2_max: *p* = 0.202

Cycling vs. Running

For the cycling vs. running direction, the Friedman test indicated a statistically significant but small overall effect of training intensity [*χ*^2^ (2) = 6.17, *p* = 0.046, Kendall's *W* = 0.05].

However, none of the *post-hoc* pairwise comparisons reached statistical significance:

- Low-intensity vs. Threshold: *p* = 0.15
- Low-intensity vs. VO_2_max: *p* = 0.79
- Threshold vs. VO_2_max: *p* = 0.07

Overall, significant effects were observed primarily for transfer direction and training intensity, whereas most group comparisons remained non-significant.

## 4 Discussion

The present study investigated how endurance athletes and coaches perceive and apply cross-training between running and cycling. Overall, the findings indicate that cross-training was not perceived as a simple or symmetrical substitution between modalities, but rather as a direction-dependent adjustment of training duration, frequency, and intensity.

### 4.1 Transfer direction

A central finding of the study was the clear directional difference in perceived transferability between modalities. Participants consistently rated running-to-cycling transfer as more practical and transferable than cycling-to-running transfer, suggesting that cycling may generally be perceived as a more acceptable substitute for running than vice versa. Similar tendencies were also reflected in the observations by Sandbakk et al. (21), who interviewed twelve highly successful Norwegian world-class endurance coaches responsible for more than 380 international medals across sports such as distance running, road cycling, swimming, triathlon, rowing, and cross-country skiing. These coaches reported that cross-training was used considerably more frequently in running sports than in cycling, indicating that modality substitution may be perceived as more relevant or practically useful within running contexts.

Participants also consistently estimated that substantially longer cycling durations were required to replace regenerative running sessions, whereas comparatively shorter running durations were considered sufficient to replace cycling sessions. This asymmetry was evident across both the Likert-scale ratings and the calculated equivalence factors. As the present study assessed perceived transferability rather than physiological responses, the following explanations should be regarded as possible interpretations of these perceptions rather than direct evidence of the underlying mechanisms. One possible explanation for this perception is that running may involve a larger active muscle mass and greater cardiovascular strain compared to cycling, even at comparable submaximal intensities (22). Furthermore, Bijker et al. (23) reported substantially greater delta efficiency during running than cycling, which may further contribute to the perception that running elicits a stronger overall training stimulus. Alongside this, prolonged running has been shown to induce greater central fatigue and reductions in maximal strength than cycling (2). Together, these physiological differences may have influenced participants’ perceptions that running provides a stronger overall training stimulus, thereby contributing to the view that longer cycling durations are required to achieve comparable training effects (7). However, these mechanisms were not directly assessed in the present study. In line with this interpretation, Millet et al. (2) suggested that physiological training transfer may be greater from running to cycling than vice versa. Cycling may simultaneously be perceived as allowing training duration to be extended more easily due to lower cardiovascular strain and reduced mechanical loading, while still maintaining aerobic training volume (3).

Importantly, the present findings further suggested that equivalence relationships between running and cycling were not linear (Supplementary Tables 8, 9). The calculated equivalence factors systematically changed across training durations, suggesting that practitioners may not perceive cross-training between modalities as adequately represented by fixed conversion ratios. Shorter running sessions were associated with higher equivalence factors when replaced by cycling, whereas the relative differences between modalities decreased with increasing duration. This finding may be particularly relevant in applied endurance training contexts, as it challenges the common practical assumption that endurance modalities can be substituted using constant time-based conversion factors. Although developed in the context of triathlon training load quantification, the fixed sport-specific factor proposed by Cejuela-Anta and Esteve-Lanao (24), in which training load is expressed relative to running (=1) and cycling is assigned a constant factor of 0.5 based on considerations such as energy cost, delayed muscle soreness, training density, and technical demands, appears inconsistent with the present findings, as perceived equivalence varied systematically across training durations rather than remaining constant.

The observed non-linear relationships may reflect differences in perceived recovery demands, cumulative mechanical stress, or training density across shorter and longer sessions (21). It is also possible that participants implicitly considered practical coaching aspects such as fatigue management, scheduling constraints, or cumulative weekly load when estimating equivalent durations. This interpretation aligns with observations from endurance coaches, who consider duration and kind of training, rating of perceived exertion, and subjective athlete feedback as essential for daily training regulation rather than relying solely on objective training quantification (25). Furthermore, Sandbakk et al. (21), emphasized that training decisions are shaped by sport-specific constraints such as mechanical and muscular loading, intensity distribution, training organization, and recovery demands rather than fixed training prescriptions. The stabilization of equivalence factors at longer durations further suggests that perceived transferability between modalities may become more consistent during prolonged low-intensity exercise.

### 4.2 Intensity

Another important finding concerned training intensity. Low-intensity training was consistently perceived as more transferable between modalities than threshold- or VO_2_max-oriented training. Particularly for the running-to-cycling direction, transferability ratings decreased substantially with increasing intensity. These findings are consistent with the principle of training specificity (26), suggesting that training adaptations become increasingly exercise-mode specific as intensity increases. One possible explanation is that low-intensity training primarily targets general aerobic capacity, which may transfer relatively well between running and cycling. This interpretation is supported by Medelli et al. (27), who found comparable heart rate responses at the aerobic threshold but clear modality-specific differences at the anaerobic threshold between cycling and running in well-trained triathletes, suggesting that physiological responses become increasingly exercise-mode specific as intensity increases. In contrast, higher-intensity sessions likely depend more strongly on sport-specific movement patterns, muscle recruitment, and physiological regulation to achieve the intended training stimulus (2). However, as no physiological variables were measured in the present study, these explanations should be regarded as possible interpretations of participants’ perceptions rather than direct evidence of the underlying mechanisms.

This may be particularly relevant for threshold-oriented training. Previous work suggests that submaximal physiological markers such as the anaerobic threshold may partly depend on the amount and type of recruited muscle mass as well as the oxidative capacity of the involved musculature (2, 28). Supporting this interpretation, Mazzeo and Marshall (29) reported that trained cyclists achieved similar VO_2_max values during cycling and treadmill running, whereas lactate and ventilatory thresholds occurred at a lower percentage of VO_2_max in the exercise mode in which athletes had not trained extensively. This may indicate that threshold-related adaptations are more exercise-mode specific than maximal aerobic capacity, potentially explaining why threshold-oriented sessions tended to be perceived as less transferable than VO_2_max-oriented training. This interpretation is supported by Bouckaert et al. (30), who observed that blood lactate responses during incremental exercise differed substantially between running and cycling, with greater discrepancies occurring when athletes were tested in a modality they had not trained extensively, further indicating that threshold-related physiological responses may be highly exercise-mode specific. These physiological findings are consistent with participants’ perceptions but cannot confirm the mechanisms underlying the observed ratings.

This interpretation is further supported by the strong agreement among participants that cycling intensity should be regulated using cycling-specific rather than running-specific heart rate zones. Such perceptions align with findings by Röcker et al. (7), who reported modality-specific differences in heart rate at the individual anaerobic threshold (IAT), with values between running and cycling differing by up to approximately 20 beats per minute. Taken together, these findings suggest that practitioners do not perceive training intensity to be directly transferable between modalities and instead view higher-intensity training as increasingly sport-specific.

The findings further suggest that cross-training may be particularly suitable for maintaining aerobic training volume while reducing sport-specific mechanical stress. This interpretation aligns with common practical applications of cross-training during periods of injury prevention, rehabilitation, or high cumulative training load (31). In such situations, low-impact modalities such as cycling are often incorporated to maintain endurance adaptations while limiting repetitive musculoskeletal loading associated with running.

### 4.3 Group differences

Group differences between athletes, coaches, and athlete–coach individuals were generally small, with significant differences emerging only for selected short-duration replacement scenarios and some directional comparisons. This finding is consistent with previous research suggesting a generally high agreement between coaches and athletes in the perception of training. In their systematic review and meta-analysis, Inoue et al. (15) reported no significant differences between coaches and athlete's ratings of overall perceived exertion (RPE) and session-RPE, nor for moderate- and high-intensity exercise. Significant discrepancies were identified only for low-intensity training, indicating that coach and athlete perceptions may diverge under specific training conditions rather than systematically across all training intensities.

The present findings extend these observations to the context of cross-training between running and cycling. Whereas previous studies primarily examined perceptions of completed training sessions, the present study assessed participants general knowledge and practical experience regarding the transferability of physiological adaptations and equivalent training durations between both exercise modalities. Such judgments are likely based on accumulated coaching and training experience rather than the perception of a single exercise bout. Given that athletes and coaches in endurance sports are commonly exposed to similar training principles and practical decision-making processes, it is plausible that both groups develop a shared understanding of how running and cycling can be substituted. However, the absence of significant group differences should be interpreted with caution, as the relatively small sample size may have limited the statistical power to detect subtle differences between groups. Future studies with larger samples are warranted to confirm whether the observed similarities reflect true agreement or insufficient power to detect small effects.

### 4.4 Limitations

The present study has several limitations that should be considered when interpreting the findings. First, the relatively small sample size limits the statistical power of the analyses and the generalizability of the findings. Therefore, the study should be regarded as exploratory. A completion rate of 57% (55 of 97 initiators) was observed. As no data were collected from non-completers, potential systematic differences between completers and non-completers (e.g., in terms of experience, sport discipline, or role) cannot be ruled out, which may limit the representativeness of the sample. In addition, participation was voluntary, introducing the possibility of self-selection bias, as individuals with a particular interest or experience in cross-training may have been more likely to participate.

Furthermore, the sample consisted predominantly of triathletes (42 of 55 participants). As triathletes routinely integrate running and cycling within their training, they may possess particularly extensive practical experience with cross-training between both modalities. However, their perceptions of transferability may differ from those of athletes and coaches operating exclusively within running or cycling environments. Consequently, the findings should be generalized to single-sport endurance populations with caution. Moreover, participants were recruited exclusively from German-speaking endurance sport settings, which may limit the cultural and contextual generalizability of the findings to athletes and coaches from other countries or sporting systems.

Another important limitation is that the study assessed subjective perceptions and practical estimations rather than physiological responses or experimentally verified transfer effects. Consequently, the findings should be interpreted as reflecting practitioners’ beliefs and accumulated experience rather than objective evidence of training effectiveness. Future experimental studies are needed to determine whether these perceptions correspond to actual physiological transfer effects between running and cycling.

Despite these limitations, the study provides systematic insight into how cross-training between running and cycling is perceived and implemented in applied endurance sport settings. Future research should integrate experimental and applied approaches to better understand how cross-training can be optimized in real-world endurance training. Particular attention should be given to the standardized quantification of training load across endurance modalities, as recent evidence highlights that inconsistent assessment of training frequency, intensity, duration, and exercise type may hinder valid comparisons between sports and the evaluation of cross-training interventions (32).

## 5 Conclusion

This study suggests that practitioners perceive cross-training between running and cycling as perceived as direction-dependent, non-linear, and regulated by coordinated adjustments of multiple training variables. Rather than representing a simple one-to-one substitution, practitioners perceived replacement between both modalities as requiring systematic modifications in training duration, intensity, and frequency, with these adjustments depending on the direction of substitution and the intended training purpose.

The observed asymmetry between running-to-cycling and cycling-to-running substitutions, together with the reduced perceived transferability of higher training intensities, indicates that practitioners generally consider cross-training to be most applicable for maintaining general aerobic adaptations rather than replacing sport-specific training demands. Overall, the findings provide a more differentiated understanding of how modality substitution is conceptualized in endurance training practice from the perspective of practitioners. However, these conclusions are based on perceived transferability rather than experimentally verified physiological responses and should therefore not be interpreted as evidence of physiological equivalence between running and cycling. The present findings may provide a foundation for future studies examining whether these practitioner perceptions correspond to physiological training adaptations.

## Data Availability

The original contributions presented in the study are included in the article/Supplementary Material, further inquiries can be directed to the corresponding author.
