# Supplementary material for: Cross-training between running and cycling: practitioner perspectives on transferability and exercise substitution
Source: Front Sports Act Living. 2026 Aug 25;8:1930728. doi: 10.3389/fspor.2026.1930728 (PMC13550123; doi:10.3389/fspor.2026.1930728)
Supplement: Supplementary file 1 [file Table1.docx]

# **Supplementary Material**

### **1:**

Table 5: Calculated equivalence factors

| Faktor | N | mean | median | SD | IQR | min | max |
| --- | --- | --- | --- | --- | --- | --- | --- |
| F1_L30_to_Bike | 55 | 2.01 | 2.00 | 0.46 | 0.00 | 1.33 | 4.00 |
| F2_L60_to_Bike | 55 | 1.81 | 2.00 | 0.35 | 0.50 | 1.17 | 3.00 |
| F3_L120_to_Bike | 55 | 1.68 | 1.75 | 0.52 | 0.50 | 0.00 | 3.00 |
| F4_L180_to_Bike | 55 | 1.57 | 1.67 | 0.55 | 0.67 | 0.00 | 3.00 |
| F5_B45_to_Run | 55 | 0.57 | 0.56 | 0.20 | 0.22 | 0.33 | 1.78 |
| F6_B60_to_Run | 55 | 0.69 | 0.50 | 0.50 | 0.25 | 0.33 | 4.17 |
| F7_B120_to_Run | 55 | 0.49 | 0.50 | 0.13 | 0.00 | 0.00 | 0.75 |
| F8_B180_to_Run | 55 | 0.48 | 0.50 | 0.15 | 0.08 | 0.00 | 0.81 |

# **2:**

Table 6: Assessment the consistency of estimations within groups

| Role | Factor | SD | IQR | Range |
| --- | --- | --- | --- | --- |
| Athlete | F1_L30_to_Bike | 0.53 | 0.00 | 2.50 |
| Athlete | F2_L60_to_Bike | 0.35 | 0.33 | 1.67 |
| Athlete | F3_L120_to_Bike | 0.56 | 0.50 | 2.96 |
| Athlete | F4_L180_to_Bike | 0.64 | 0.63 | 3.00 |
| Athlete | F5_B45_to_Run | 0.28 | 0.22 | 1.44 |
| Athlete | F6_B60_to_Run | 0.74 | 0.17 | 3.83 |
| Athlete | F7_B120_to_Run | 0.13 | 0.00 | 0.75 |
| Athlete | F8_B180_to_Run | 0.16 | 0.06 | 0.67 |
| Coach | F1_L30_to_Bike | 0.31 | 0.50 | 1.17 |
| Coach | F2_L60_to_Bike | 0.34 | 0.67 | 0.83 |
| Coach | F3_L120_to_Bike | 0.51 | 0.75 | 2.00 |
| Coach | F4_L180_to_Bike | 0.48 | 0.56 | 2.00 |
| Coach | F5_B45_to_Run | 0.10 | 0.22 | 0.22 |
| Coach | F6_B60_to_Run | 0.11 | 0.23 | 0.25 |
| Coach | F7_B120_to_Run | 0.13 | 0.08 | 0.50 |
| Coach | F8_B180_to_Run | 0.16 | 0.11 | 0.64 |
| Athlete_ Coach | F1_L30_to_Bike | 0.41 | 0.00 | 1.50 |
| Athlete_ Coach | F2_L60_to_Bike | 0.24 | 0.38 | 0.50 |
| Athlete_ Coach | F3_L120_to_Bike | 0.27 | 0.50 | 0.58 |
| Athlete_ Coach | F4_L180_to_Bike | 0.39 | 0.63 | 1.17 |
| Athlete_ Coach | F5_B45_to_Run | 0.14 | 0.22 | 0.33 |
| Athlete_ Coach | F6_B60_to_Run | 0.12 | 0.23 | 0.25 |
| Athlete_ Coach | F7_B120_to_Run | 0.09 | 0.06 | 0.33 |
| Athlete_ Coach | F8_B180_to_Run | 0.10 | 0.04 | 0.33 |

# **3:**

Table 7: Practically applicable equivalence values between regenerative running and cycling sessions

| Question | N | mean | median | SD | IQR | min | max |
| --- | --- | --- | --- | --- | --- | --- | --- |
| Q1_L30_to_Bike | 55 | 60.2 | 60 | 13.8 | 0 | 40 | 120 |
| Q2_L60_to_Bike | 55 | 108.4 | 120 | 20.8 | 30 | 70 | 180 |
| Q3_L120_to_Bike | 55 | 201.2 | 210 | 62.7 | 60 | 0 | 360 |
| Q4_L180_to_Bike | 55 | 282.4 | 300 | 99.7 | 120 | 0 | 540 |
| Q5_B45_to_Run | 55 | 25.6 | 25 | 8.9 | 10 | 15 | 80 |
| Q6_B60_to_Run | 55 | 39.5 | 30 | 29.7 | 15 | 20 | 250 |
| Q7_B120_to_Run | 55 | 58.7 | 60 | 15.0 | 0 | 0 | 90 |
| Q8_B180_to_Run | 55 | 86.5 | 90 | 26.8 | 15 | 0 | 145 |

# **4:**

Table 8: Relationships between running and cycling (run to bike)

|  | Group 1 | Group 2 | N1 | N2 | Statistic | p | p.adj | p.adj.signif |
| --- | --- | --- | --- | --- | --- | --- | --- | --- |
| Faktor | F1_L30_to_Bike | F2_L60_to_Bike | 55 | 55 | 320.5 | < 0.001 | < 0.001 | **** |
| Faktor | F1_L30_to_Bike | F3_L120_to_Bike | 55 | 55 | 478.0 | < 0.001 | < 0.001 | **** |
| Faktor | F1_L30_to_Bike | F4_L180_to_Bike | 55 | 55 | 712.5 | < 0.001 | < 0.001 | **** |
| Faktor | F2_L60_to_Bike | F3_L120_to_Bike | 55 | 55 | 178.5 | 0.029 | 0.034 | * |
| Faktor | F2_L60_to_Bike | F4_L180_to_Bike | 55 | 55 | 489.0 | 0.001 | 0.003 | ** |
| Faktor | F3_L120_to_Bike | F4_L180_to_Bike | 55 | 55 | 348.5 | 0.017 | 0.034 | * |

Table 9: Relationships between running and cycling (bike to run)

|  | Group 1 | Group 2 | N1 | N2 | Statistic | p | p.adj | p.adj.signif |
| --- | --- | --- | --- | --- | --- | --- | --- | --- |
| Faktor | F5_B45_to_Run | F6_B60_to_Run | 55 | 55 | 90 | < 0.001 | < 0.001 | **** |
| Faktor | F5_B45_to_Run | F7_B120_to_Run | 55 | 55 | 1062 | < 0.001 | 0.002 | ** |
| Faktor | F5_B45_to_Run | F8_B180_to_Run | 55 | 55 | 891 | 0.002 | 0.004 | ** |
| Faktor | F6_B60_to_Run | F7_B120_to_Run | 55 | 55 | 528 | < 0.001 | < 0.001 | **** |
| Faktor | F6_B60_to_Run | F8_B180_to_Run | 55 | 55 | 711 | < 0.001 | < 0.001 | **** |
| Faktor | F7_B120_to_Run | F8_B180_to_Run | 55 | 55 | 260 | 0.363 | 0.363 | ns |
